# AARS1 and AARS2: From Protein Synthesis to Lactylation-Driven Oncogenesis

**DOI:** 10.3390/biom15091323

**Published:** 2025-09-16

**Authors:** Lingyue Gao, Jihua Guo, Rong Jia

**Affiliations:** 1 State Key Laboratory of Oral & Maxillofacial Reconstruction and Regeneration, Key Laboratory of Oral Biomedicine Ministry of Education, Hubei Key Laboratory of Stomatology, School & Hospital of Stomatology, Wuhan University, Wuhan 430072, China; gao_lingyue@whu.edu.cn; 2 Department of Endodontics, School & Hospital of Stomatology, Wuhan University, Wuhan 430072, China

**Keywords:** AARS1, AARS2, lactylation, cancer therapy

## Abstract

Aminoacyl-tRNA synthetases (AARSs), traditionally recognized for their essential role in protein synthesis, are now emerging as critical players in cancer pathogenesis through translation-independent functions. Lactate-derived lactylation, a post-translational modification, plays an increasingly important role in tumorigenesis in the context of high levels of lactate in tumor cells due to the Warburg effect. Current research has highlighted AARS1/2 as lactate sensors and lactyltransferases that catalyze global lysine lactylation in cancer cells and promote cancer proliferation, providing a new perspective for cancer therapy. This review synthesizes the canonical and non-canonical functions of AARS1/2, with a particular focus on their lactylation-related mechanisms; details how lactylation acts as a mechanistic bridge linking AARS1/2 to diverse oncogenic signaling pathways, thereby promoting cancer hallmarks such as metabolic reprogramming, uncontrolled proliferation, immune escape, and therapy resistance; and proposes strategies to target AARS1/2 or modulate relative lactylation, offering a potential avenue to translate these insights into effective cancer therapies.

## 1. Introduction

Aminoacyl-tRNA synthetases (AARSs) are enzymes that covalently bind a specific amino acid to the corresponding tRNA, playing critical roles in mRNA translation [1]. AARSs are classified into two distinct groups (Classes I and II) based on the evolutionarily conserved structural folds of their catalytic domains (the Rossmann fold for Class I and the antiparallel β-fold for Class II). Each class possesses unique, conserved sequence motifs that are integral to their distinct active site configurations and catalytic mechanisms [2,3,4].

Alanyl-tRNA synthetases 1/2 (AARS1/2), belonging to Class II of the AARS family, are enzymes that play crucial roles in protein synthesis by catalyzing the attachment of the amino acid alanine to its cognate tRNA [1,5]. Additionally, AARSs exhibit diverse translational and non-translational functions, and they are associated with numerous genetic disorders [6]. AARS1 and AARS2 have been identified as regulatory hubs, participating in diverse cellular processes through lactylation-dependent mechanisms in multiple physiological and pathological contexts. For example, AARS1 promoted osteoblast differentiation via lactylation of lysine 230 of Osterix (Osx), a key transcription factor in osteoblast differentiation, which enhanced Osx interaction with WD repeat domain 5 (WDR5), facilitating H3K4me3 enrichment on target gene promoters and activating their transcription [7]. In an intestinal ischemia–reperfusion mouse model, an elevated level of AARS2 mediated K18 lactylation of histone H3 on the acyl-CoA synthetase long-chain family member 4 (ACSL4) promoter, activating its expression and subsequent cell ferroptosis [8]. During premature ovarian insufficiency, AARS2-mediated lactylation and inactivation of carnitine palmitoyl transferase 2 (CPT2) promoted granular cell proliferation and primordial follicle development by activating peroxisome proliferator-activated receptor γ (PPARγ)–follicle-stimulating hormone (FSH) synergy [9]. In mice cardiomyocytes, AARS2 overexpression enhanced the protein translation of pyruvate kinase M2 (PKM2) and the ratio of PKM2 dimers to tetramers that promote glycolysis, inhibiting cardiomyocyte apoptosis and mitochondrial reactive oxide species production and shifting cellular metabolism from oxidative phosphorylation to glycolysis, which consequently promoted cardiomyocyte survival from ischemia and hypoxia stress [10].

Beyond that, the roles of AARSs in cancer progression are emerging. For example, AARSs have been reported to sense the sufficiency of their cognate amino acid by catalyzing lysine aminoacylation (K-AA) of its target proteins, hence regulating cellular processes and contributing to tumorigenesis [11,12]. AARS1 and AARS2 are generally upregulated in many cancers, consistent with increased protein synthesis demand. AARS2 is frequently amplified across multiple cancers, including lung adenocarcinoma, lung squamous cell carcinomas, and breast invasive carcinoma, suggesting its potential pro-tumorigenic role [13].

Historically, the evolution of cancer therapy has progressed from non-specific cytotoxic agents to molecularly targeted therapies, reflecting a deeper understanding of tumor biology [14]. This progression underscores the importance of identifying and validating novel mechanistic drivers of oncogenesis. Lysine lactylation mediated by AARS1/2 has emerged as a direct molecular link between cellular metabolism and oncogenic signaling [15,16], which could serve as novel biomarkers for cancer treatment response. The integration of biomarkers into drug development has consistently been shown to significantly improve clinical trial success rates and patient outcomes [17].

In this review, we summarize the latest research progress on the structure, expression, and function of AARS1/2, especially focusing on their lactylation-related enzyme activity and their roles in cancer; we summarize relevant signaling pathways in processes associated with cancer progression; and we discuss potential strategies to block cancer progression by targeting AARS1/2.

## 2. Canonical Functions of AARS1 and AARS2 Concerning Protein Synthesis

### 2.1. The Structure and Functional Domains of AARS1 and AARS2

The human AARS1 (also called AlaRS, CMT2N, or AARS) gene is located at 16q22.1, and it contains 21 exons and encodes a 968-amino acid (aa) protein. The AARS1 protein has a catalytic domain, a tRNA-binding domain, an editing domain, and a C-terminal domain. A nuclear localization motif (aa 750–763) was found in the C-terminal domain [16]. Two AARS1 molecules form a homodimer through the C-terminal region [18]. Even though the dimerization of AARS1 is not required for aminoacylation, its role in lactylation remains unknown. According to its record in Genbank, the AARS1 gene may produce a variant without exon 15 and encode a truncated protein with a distinct C-terminal. Two additional in-frame splice variants were also reported [19]. The AARS1 protein sequence is highly conserved from fish to humans (Figure S1), indicating its key role during evolution.

**Figure 1:**
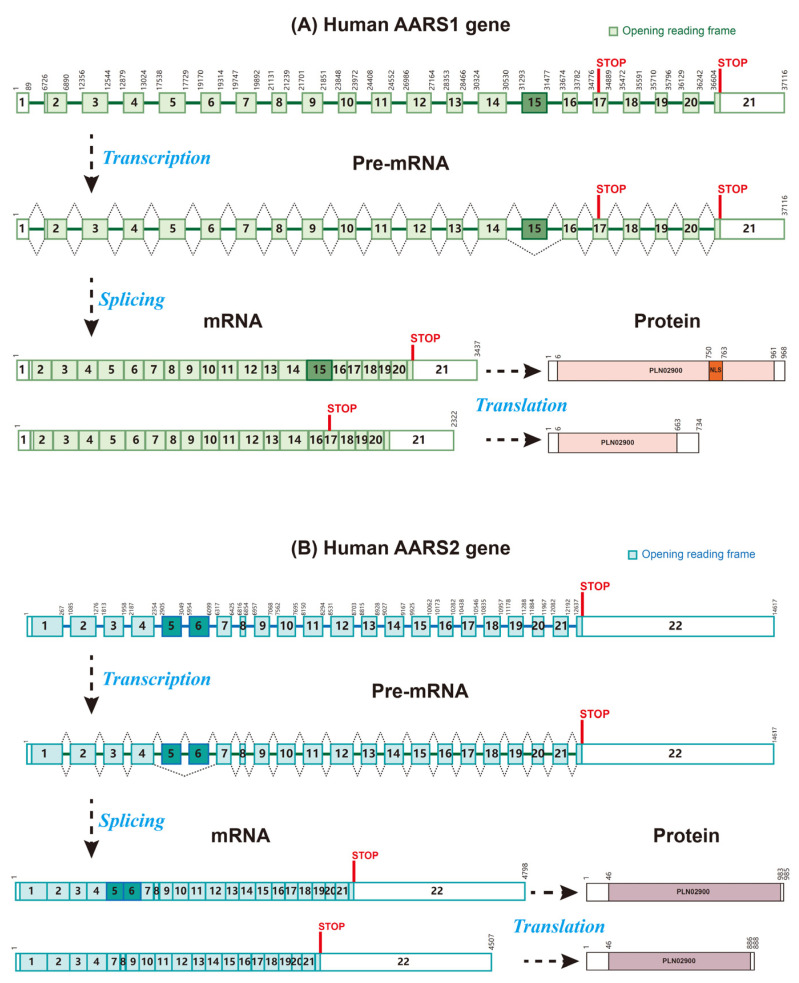
Gene, mRNA, and protein domain organization of human AARS1 and AARS2. (**A**) The human AARS1 gene contains 21 exons and 20 introns and expresses 2 mRNA isoforms. The predicted isoform is generated from the skipping of exon 15, bearing a pre-mature stop codon on exon 17. The full-length AARS1 protein (968 amino acid residues) is encoded by transcripts with exon 15, whereas the transcripts with exon 15 skipped encode a truncated AARS1 protein (735 amino acid residues). (**B**) The human AARS2 gene contains 22 exons and 21 introns and expresses 2 mRNA isoforms. The full-length AARS2 protein (985 amino acid residues) is encoded by transcripts with 22 exons. The shorter isoform is generated from the skipping of exons 5 and 6, encoding a shorter protein (888 amino acid residues) missing a 97 aa middle part.

The human AARS2 (also called AARSL, MTALARS, LKENP, or COXPD8) gene is located at 6p21.1, and it contains 22 exons and encodes a 985 aa protein. A variant with in-frame deletions of exons 5 and 6 encodes a short protein missing a 97 aa middle part. The human AARS2 protein shares a 43.3% amino acid sequence identity with the human AARS1 protein, as well as four domains. However, the AARS2 protein has no nuclear localization motif and is located in mitochondria. The protein sequence of AARS2 is much less conserved than that of AARS1 during evolution. However, the residues binding to lactate are highly conserved between different species (Figure S2), indicating that it is an evolutionarily conserved sensor of lactate (Figure 1).

### 2.2. The Mechanism of Aminoacylation, Including Substrate Specificity (Alanine) and tRNA Charging

AARSs follow a universal two-step reaction during aminoacylation, powered by adenosine triphosphate (ATP) [20]:

Step 1 (amino acid activation): ATP + amino acid → aminoacyl-adenylate (aa-AMP) + inorganic pyrophosphate (PPi). The α-carboxylate oxygen of the amino acid attacks the α-phosphorus of Mg-ATP, forming a mixed anhydride linkage.

Step 2 (tRNA charging): aa-AMP + tRNA → aa-tRNA + AMP. Either the 2′-OH (Class I) or 3′-OH (Class II, except for PheRS) of the tRNA’s 3′-terminal A76 nucleotide attacks the carbonyl carbon of the adenylate, generating aminoacyl-tRNA, along with the release of AMP.

Substrate specificity in aminoacylation features steric/chemical discrimination at synthetic sites with proofreading via editing domains. The alanyl-tRNA synthetase exemplifies imperfect synthetic site discrimination due to a shallow binding pocket, which fails to exclude serine due to its similar size and relies heavily on *cis*- and *trans*-editing pathways to prevent mistranslation [21,22].

### 2.3. Heterozygous Mutations of AARS1/2 Impair Their Function

Heterozygous mutations of AARS1 and AARS2 cause multi-organ disorders via disrupted protein synthesis or tRNA charging. Whole-exome/genome sequencing of unsolved non-photosensitive trichothiodystrophy (NPS-TTD) cases revealed compound heterozygous *AARS1* mutations in two patients (TTD236AM and TTD1GL), which featured significantly reduced cellular levels of the AARS1 protein in patient fibroblasts, causing protein instability and impaired enzymatic activity [23]. A homozygous *AARS1* variant (c.1817C>T, p.Thr606Ile) was identified in a consanguineous family with adult-onset leukoencephalopathy with spheroids and pigmented glia, highlighting impaired aminoacylation efficiency and tRNA mischarging as key disease mechanisms [24]. A case of recurrent acute liver failure reported compound heterozygous missense *AARS1* variants (p.[Leu298Gln];[Arg751Gly]), exhibiting hepatocyte dysfunction through impaired protein synthesis and temperature-sensitive aminoacylation defects [25]. The cardiomyocyte-specific deletion of poly(rC) binding protein 1 (*Pcbp1*) in mice led to the aberrant skipping of AARS2 exon 16, causing a frameshift and premature termination codon, thus reducing AARS2 protein expression [26]. Impaired AARS2 expression was associated with oxidative phosphorylation disorder and congenital heart defects [26].

## 3. Lactylation: A Novel Mechanistic Link Between AARS1/AARS2, Metabolism, and Cancer

### 3.1. Cancer Metabolism and the Warburg Effect

Hypoxia is nearly a universal characteristic of solid tumors [27]. The level of oxygen in normal tissues ranges from about 3% to 7.4%; this is much lower than that in cancer cells, which ranges from 0.3% to 4.2%, with the median oxygen level being less than 2% [28]. Tumor oxygenation is dynamic, with slow periodic fluctuations (0.12–0.28 cycles/min) in microvessel hemoglobin saturation contributing to transient hypoxia [29]. There are two types of oxygen gradients in tumors: one is a radial gradient from oxygen diffusion limitations, and the other is a longitudinal gradient from a heterogeneous oxygen distribution due to abnormal vasculature, resulting in hypoxic regions compared to relatively normoxic areas near blood vessels [29,30]. Some tumors have been reported to contain two metabolically distinct subpopulations: one is hypoxic and glycolysis-dependent, secreting lactate, while the other is better oxygenated and preferentially imports this lactate as a primary fuel for oxidative phosphorylation, revealing a symbiotic relationship [31,32]. According to the Warburg effect, cancer cells can reprogram their glucose metabolism and preferentially metabolize glucose to lactate via glycolysis, even under aerobic conditions, which is inefficient for ATP production compared to oxidative phosphorylation [33,34]. Its significance extends far beyond a simple metabolic switch, as glycolytic intermediates are diverted into biosynthetic pathways, including the pentose phosphate pathway for nucleotides and the serine/glycine synthesis pathway for proteins and lipids, to facilitate cancer cell proliferation [34].

### 3.2. Introduction of Lactylation and Lactate Sensing

Lactate is commonly known as a metabolic product of glycolysis that provides energy. The Warburg effect highlights upregulated levels of intracellular lactate and acidification in the tumor microenvironment. Apart from metabolic products, lactate has been identified as a signaling molecule involved in diverse biological processes, including the immune response, angiogenesis, fibrosis, and tumor proliferation [35,36,37].

Lactate has been detected as a mediator of gene expression through histone and non-histone lactylation [38,39]. The role of lactylation in tumorigenesis has been increasingly emphasized, as it alters the protein expression of key metabolic proteins and impacts cellular processes [40,41,42]. The acetyltransferase p300 has been reported to mediate histone lactylation as a lactyltransferase, which relies on the presence of lactyl-coenzyme A (lactyl-CoA) [38]. However, the concentration of lactyl-CoA in mammalian cells is 20–350 times lower than that of predominant acyl-CoAs [43], which indicates that p300 may not largely contribute to global intracellular lactylation. Given that the chemical structure of alanine is highly similar to that of lactate, AARS1/2 have been speculated and identified as sensors and lactyltransferases of intracellular lactate. They sense high levels of lactate and mediate global lysine lactylation, affecting protein function and cellular processes.

### 3.3. AARS1 and AARS2 Sense Lactate and Mediate Lactylation

#### 3.3.1. The Recognition of Lactate by AARS1 and AARS2

In general, AARS1 is mainly distributed in the cytoplasm [16]. There is an evolutionarily conserved nuclear localization signal (NLS) motif in the C-terminal region of AARS1. AARS1 shuttles into the nucleus on the basis of the interaction of its NLS motif with importin karyopherin subunit alpha 4 (KPNA4) [16]. In response to intracellular lactate accumulation, the interaction of the NLS in AARS1 with KPNA4 is intensified, thus enhancing the nucleus translocation of AARS1 and its downstream effects [16]. AARS2 is a mitochondrial alanyl-tRNA synthetase without an NLS motif [16,44]. AARS2 primarily senses lactate and catalyzes the global lactylation of the mitochondrial proteome [44].

A mutagenesis screen has detected residues crucial for AARSs’ binding to lactate: M46, R77, N216, D239, and G241 in AARS1 and M79, R110, N242, D265, and G267 in AARS2 [45]. Importantly, these key residues are also conserved in the alanyl-tRNA synthetase (AlaRS) (M43, R69, W170, N212, D235, and G237) of *E. coli* [15,45]. Interestingly, an R77Q mutation frequently found in patients with gastric cancer enhances the catalytic capacity of AARS1 [16], while mutating other essential lactate-binding residues to alanine simultaneously eliminates AARSs’ binding to lactate [45]. These results indicate that the residues that bind to lactate are potential target sites for controlling AARS1 activity.

Lactate exists widely in diverse normal tissues and organs, and it acts as a major energy source and signaling molecule, with a physiological range of 0.5–20 mM. Tumors often have higher levels of lactate, up to 40 mM [46]. In vitro, a supplement of 25 mM lactate can trigger AARS1 nuclear translocation and significant global lactylation. Therefore, in normal cells with low concentrations of lactate, it may be used more in the metabolism pathway than in the protein lactylation signaling pathway. Blocking the nuclear translocation of AARS1 may significantly affect oncogenic lactylation modification in tumor cells while having fewer effects on normal cell metabolism with lactate.

#### 3.3.2. Lactylation Mediated by AARS1 and AARS2

**Figure 2:**
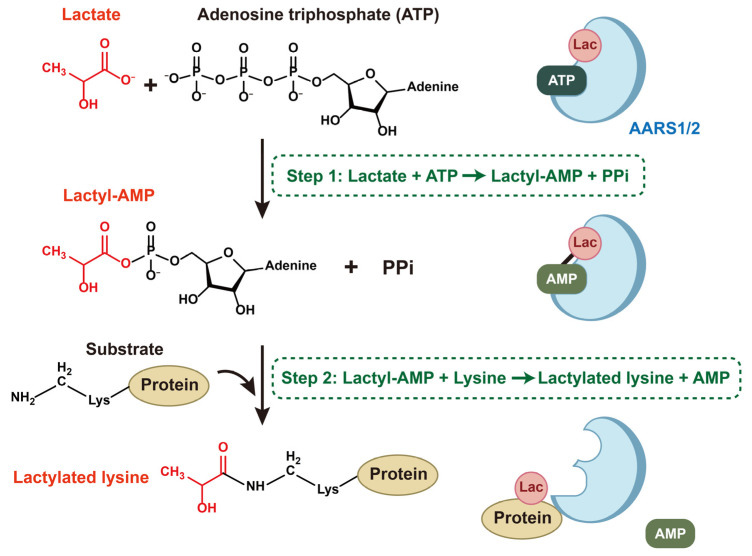
The process of AARS1/2-mediated lactylation in target proteins.

The underlying mechanism of lactylation mediated by AARS1/2 is similar to the way they bind L-alanine to lysine [44,45]. AARS1/2 catalyze the lactylation of lysine via a two-step process, utilizing lactate as a lactyl supplier and ATP as an energy source (Figure 2): First, AARSs attach to lactate and form lactate-AMP, a reactive lactyl-phosphate bond-containing intermediate product, with the consumption of ATP and the release of PPi. Second, AARS1/2 catalyze the conjugation of the lactyl group on lactyl-AMP to lysine residues on substrates, releasing AMP [11,15,16,45]. During this process, AARS1 and AARS2 have shared targets, as well as unique favored substrates and sequence motifs [45].

## 4. The Expression and Roles of AARS1 and AARS2 in Cancers

### 4.1. Expression of AARS1/2 in Cancers

AARS1 is significantly overexpressed in gastric cancer (GC) [16], duodenal cancer (DC) [47], and many other types of cancer [15], and it is negatively associated with the survival of gastric cancer [16] and breast cancer patients [15]. In addition, the expression of AARS1 increasingly enhanced during duodenal cancer progression [47]. The overexpression of AARS1 enhanced cell proliferation and invasion, whereas AARS1 knockdown suppressed these capacities in both Hutu80 and WDC-1 gastric cancer cell lines [47]. A combination survival analysis revealed that patients with a high co-expression of ULBP1, AARS1, and DDIT3 exhibited a 2.2-fold increased risk of death from colon adenocarcinoma (COAD) compared to patients with a low expression of all three genes [48].

An elevated expression of AARS2 has been highlighted among most cancers, especially colorectal cancer (CRC) [49], hepatocellular carcinoma (HCC), and COAD, and it is closely associated with poor prognosis and survival in HCC and COAD patients [50,51]. AARS2 was identified as one of the nine key nuclear mitochondrial-related genes used to construct a prognostic risk score (RS) model for COAD, in which a higher expression of AARS2 contributed to a higher RS, categorizing patients into a higher-risk group with a poorer prognosis and lower overall survival time [52].

### 4.2. AARS1/2 and Cancer Cell Proliferation and Migration

AARS1 knockdown significantly suppresses cancer cell proliferation in vitro and tumorigenesis in vivo. AARS1 expression is positively correlated with a large tumor size, lymph node metastasis, and a high tumor stage in gastric cancer, suggesting that AARS1 may promote tumor progression [16]. AARS1 catalyzes the lysine-alanylation (K-Ala) of poly (ADP-ribose) polymerase 1 (PARP1) in K621 peptides, suppressing PARP1 activity and cell apoptosis, thereby promoting tumor growth [47]. The inhibition of K-Ala with alaninol reversed the pro-tumor effect [47]. A genome-wide CRISPR screen identified AARS1 as the protein that most strongly participates in the lactylation and inactivation of p53 in HeLa cells with wild-type p53 [15]. AARS1 mediates global lysine lactylation and transduces high levels of lactate into cell proliferation signals in cancers [15,16]. The nuclear localization of wild-type AARS1 promotes the lactylation of YAP and TEAD, activating the Hippo pathway and promoting cancer cell proliferation. In contrast, an NLS-deleted mutant AARS1 failed to lactylate targets and rescue the suppression of cell proliferation and tumor formation caused by AARS1 knockout, indicating that nuclear localization is required for AARS1’s oncogenic role [16]. Moreover, lactate coordinates with AARS1 by promoting its nuclear localization [16]. AARS2 participates in the cell cycle and mTOR signaling pathway, and it motivates cell proliferation and the migration of HCC cells [50].

### 4.3. AARS1/2 and the Cancer Microenvironment

Apart from tumor cells, there are diverse cellular and non-cellular components in the tumor microenvironment (TME), including immune cells, blood vessels, and molecular signaling networks [53,54]. In macrophages, AARS2 mediates cyclic GMP–AMP synthase (cGAS) lactylation, which abolishes its DNA-binding capability and leads to the downregulation of innate immune responses [45].

### 4.4. AARS2 and Mitochondrial Respiration

AARS2 has been identified as a hub gene that regulates mtDNA reproduction at the translational level and mitochondrial respiration. The depletion of AARS2 impaired mitochondrial respiration [51]. However, the upregulation of AARS2 triggered by hypoxia lactylated pyruvate dehydrogenase A1 (PDHA1) and carnitine palmitoyltransferase 2 (CPT2), leading to their inactivation and restraining oxidative phosphorylation (OXPHOS) [44]. Given the fact that many solid tumors feature a hypoxic state and high levels of lactate due to the Warburg effect, where cancer cells prefer glycolysis over OXPHOS [55,56], it is speculated that the overexpression of AARS2 in the context of hypoxia may inhibit OXPHOS and contribute to glycolysis in cancer cells, enabling cancer cells to adapt to hypoxia.

### 4.5. AARS1/2 and Cancer Therapy Resistance

In HT1376 and RT112 cells (human bladder cancer cells), the inhibition of AARS1 suppressed the lactylation of YTH N6-methyladenosine RNA-binding protein C1 (YTHDC1) and promoted the protein levels of YTHDC1. Meanwhile, overexpressed AARS1 enhanced the lactylation of YTHDC1 and reduced the protein levels of YTHDC1. The AARS1-mediated lactylation of YTHDC1 impaired the sensitivity of bladder cells to enfortumab vedotin (EV) therapy in high-glucose contexts [57]. Additionally, AARS1 was identified as the primary lactyltransferase responsible for catalyzing the lactylation of nudix hydrolase 21 (NUDT21) in esophageal squamous cell carcinoma (ESCC) cell lines (KYSE30 cells). NUDT21 lactylation conferred resistance to cuproptosis-inducing agents like elesclomol-Cu^2+^ and disulfiram-Cu^2+^ in ESCC cells and reduced ESCC cells’ sensitivity to cuproptosis [58] (Table 1). The proposition that AARS1/2-driven lactylation contributes to resistance against EV therapy and cell death inducers like cuproptosis holds significant clinical potential. Despite that, to assess its true translational meaningfulness, this mechanism must be compared to other well-characterized resistance pathways, such as target gene mutations, bypass signaling activation, and drug efflux pumps [59,60]. However, the roles of lactylation and AARS1/2 in other cancer resistance pathways are unclear, thus requiring further investigation and remaining a promising future direction. In addition, pooled CRISPR-Cas9 screening, in particular, has become a powerful tool for unbiasedly pinpointing the genetic determinants of therapeutic resistance [61]. For instance, genome-wide CRISPR screens have been integrated with cell line pharmacogenomic data to systematically identify novel genes conferring resistance to trametinib [62]. This approach highlights the significance of validating the role of AARS1/2 in lactylation-driven cancer therapy resistance. Large-scale genetic screens could specifically test whether the knockout of AARS1/2 or key lactylation targets reverses resistance to EV therapy or cuproptosis inducers.

## 5. The Signaling Pathways of Lactate and AARS1/2 in Cancers

**Figure 3:**
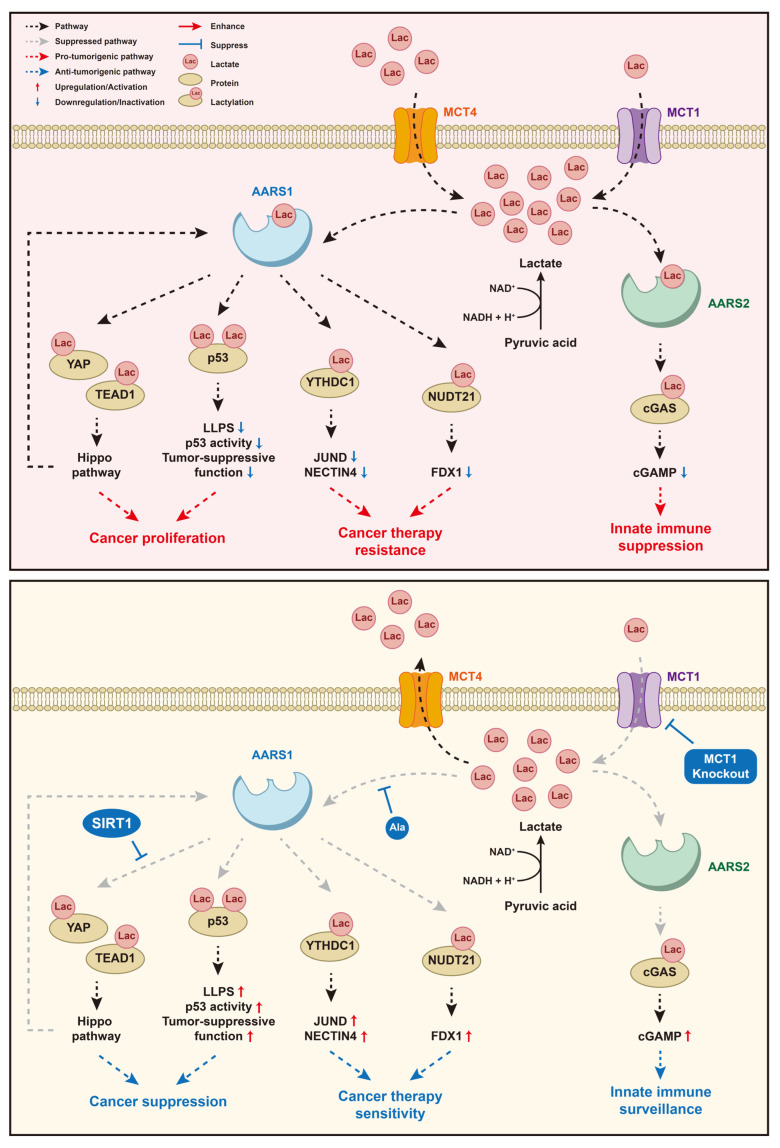
The signaling pathways of lactate mediated by AARS1 and AARS2 in cancer cells and macrophages, including lactate/AARS1/p53, lactate/AARS1/YAP&TEAD1, lactate/AARS1/YTHDC1, lactate/AARS1/NUDT21, and lactate/AARS2/cGAS.

Lactate sensors link the metabolite lactate generated from the Warburg effect to the downstream signal of tumor cell proliferation. The lactate signaling pathway is an important physiological process in tumorigenesis, which involves lactate sensors like AARSs and various downstream substrates, combining tumor cell metabolism with proteomic alterations (Figure 3).

### 5.1. Lactate/AARS1/p53

Lactate accumulation from tumors can be sensed by AARS1 and transferred into the lactylation of p53, resulting in p53 inactivation and weakened p53 tumor-suppressing function [15]. AARS1 mediates the site-specific lactylation of p53, whose lysine 120 (K120) and lysine 139 (K139), located in the DNA-binding domain, are lactated. As a result, the AARS1-mediated lactylation of p53 impairs its ability to bind DNA containing p53-response elements and disrupts its liquid–liquid phase separation (LLPS), a process essential for its transcriptional activation and formation of nuclear condensates upon genotoxic stress. Lactylated p53 showed reduced transcriptional activity toward target genes such as PUMA and p21, which are critical for apoptosis and cell cycle arrest in response to DNA damage [15]. Moreover, p53 lactylation may competitively occupy the hydroxyl group on lysine and antagonize p53 acetylation, thus inhibiting p53 activation [15]. AARS1 overexpression and p53 lactylation are closely related to poor prognosis in cancer patients [15]. In animal models, β-alanine antagonizes lactate binding to AARS1, reducing p53 lactylation and attenuating tumorigenesis [15]. However, somatic p53 mutations and inactivation frequently occur in almost every type of cancer [64,65]. The AARS1-p53 lactylation axis depends on wild-type p53 and may not be applicable in p53-mutated cancers.

### 5.2. Lactate/AARS1/YAP&TEAD1

Analogously, in gastric cancer, AARS1 is translocated into the nucleus in response to intracellular lactate accumulation and directly lactylates the Yes1-associated transcriptional regulator (YAP) at the K90 residue and TEA domain transcription factor 1 (TEAD1) at the K108 residue, activating the expression of Hippo pathway target genes and thus promoting tumor cell proliferation [16]. As AARS1 itself is one of the downstream target genes, AARS1 and YAP-TEAD1 form a positive feedback loop in the context of lactate during tumor progression [16]. The overexpression of sirtuin 1 (SIRT1), belonging to the sirtuin family of NAD^+^-dependent deacetylases, can suppress the lactylation of YAP and TEAD1 [16]. L-alanine can competitively bind to the same site as lactate on AARS1 and act as an inhibitor of AARS1’s lactyltransferase activity [16].

### 5.3. Lactate/AARS1/YTHDC1

Lactate overproduction during hyperglycemia promoted the AARS1-mediated lactylation of YTHDC1 at lysine 82 (K82), which enhanced the binding of E3 ubiquitin ligase ring finger protein 183 (RNF183) to YTHDC1, leading to the ubiquitination and proteasomal degradation of YTHDC1 in bladder cancer (BC) cell lines (HT1376 and RT112 cells). Reduced YTHDC1 led to the destabilization of JunD proto-oncogene (JUND) mRNA in an m^6^A-dependent manner and impaired JUND protein levels, resulting in reduced transcription of its target gene nectin cell adhesion molecule 4 (NECTIN4), whose decreased expression ultimately reduced BC cell sensitivity to EV therapy [57]. β-alanine, a lactate analog that inhibited AARS1-mediated lactylation, enhanced EV sensitivity [57]. Therefore, AARS1 is highly associated with the reduced sensitivity of BC cells to EV therapy in high-glucose contexts [57].

### 5.4. Lactate/AARS1/NUDT21

In esophageal squamous cell carcinoma (ESCC) cell lines, AARS1 lactylated NUDT21 at lysine 23 (K23), which enhanced NUDT21’s interaction with cleavage and polyadenylation specific factor 6 (CPSF6), forming the CFIm complex and promoting distal polyadenylation site (dPAS) selection in ferredoxin 1 (FDX1) pre-mRNA [58]. The usage of the distal polyadenylation site produces a long 3′ UTR isoform of FDX1 and reduces *FDX1* mRNA stability and protein expression, which desensitizes ESCC cells to cuproptosis induced by copper ionophores [58]. The AARS1-NUDT21-CPSF6-FDX1-cuproptosis axis linked metabolic reprogramming to post-transcriptional regulation and therapy resistance. Inhibiting AARS1 activity genetically or with β-alanine or reducing lactate production with oxamate or the clinical lactate dehydrogenase (LDHA) inhibitor stiripentol sensitized ESCC cells to cuproptosis [58].

### 5.5. Lactate/AARS2/cGAS

A high level of intracellular lactate helps build an immunosuppressive microenvironment that benefits tumor progression through the lactylation of cyclic GMP–AMP synthase (cGAS) [66,67]. In vitro, AARS1/2 directly lactylates cGAS at the N-terminus at K131 in humans and K156 in mice, respectively [45]. In vivo, AARS2 mediates the lactylation of cGAS [45]. The lactylation of specific lysine at cGAS’s N-terminus damages its liquid–liquid phase separation (LLPS) and enzyme activity, as well as DNA-sensing function, inhibiting the synthesis of cyclic GMP–AMP and innate immune responses [45]. Blocking monocarboxylate transporter 1 (MCT1), a proton-dependent transporter facilitating lactate influx, to prevent innate immune cells from intracellular lactate accumulation is a possible way to suppress immune evasion and tumorigenesis [45].

## 6. AARS1 and AARS2 as Potential Therapeutic Targets for Cancers

### 6.1. Small-Molecule Inhibitors of Catalytic Activity

Several evolutionarily conserved residues that are critical for lactate binding have been identified in human AARS1 (M46, R77, N216, D239, and G241), AARS2 (M79, R110, N242, D265, and G267), and *E. coli* AlaRS (M43, R69, W170, N212, D235, and G237) [16,17]. Mutation of one or two of the residues listed above to alanine undermines lactate binding, and mutation of those residues simultaneously clears off lactate binding [15,45]. Meanwhile, R77Q mutation in AARS1 has been detected evidently in gastric cancer and, in fact, promotes the enzymatic capacity of AARS1 [16]. Therefore, it is a potential therapeutic strategy to precisely identify the active residues crucial for lactylation in AARS1/2, figure out the specific role that each residue plays, and develop small-molecule manipulators to regulate AARSs’ lactyltransferase activity.

### 6.2. Antagonizing Protein–Protein Interactions Involved in Lactylation

Considering the analogical structure between L-alanine and L-lactate, AARS1/2 shift from functioning as alanyl-tRNA synthetases to lactyltransferases. In the context of the TME, the concentration of intracellular lactate rises due to the Warburg effect, thus propelling AARS1/2 toward catalyzing oncogenic lactylation. Studies have shown that L-alanine competitively antagonizes the lactate-binding pocket on AARS1/2, attenuating AARS-mediated lactylation and tumor progression [15,16,45], as well as β-alanine [57,58]. Regulating the relative abundance of lactate versus alanine to determine the function of AARS1/2 as lactyltransferases or alanyl-tRNA synthetases is a promising way to control tumor metabolism.

### 6.3. Targeting Subcellular Localization

How AARSs play the lactylation–enzymatic role is largely dependent on their subcellular localization. The proportion of AARS1 in the nucleus increases under high levels of intracellular lactate, while AARS1 in the cytoplasm decreases [16]. Lactate strengthened the interaction of the NLS motif in AARS1 with importin KPNA4 and facilitated AARS1 shuttling into the nucleus [16]. Therefore, AARS1 is supposed to predominantly catalyze global lactylation in the nucleus. Further investigation is required upon targeting the nuclear translocation of AARS1 to reverse the tumor-promoting lactylation.

### 6.4. Modulating Lactylation Erasers to Suppress Oncogenic Lactylation

**Figure 4:**
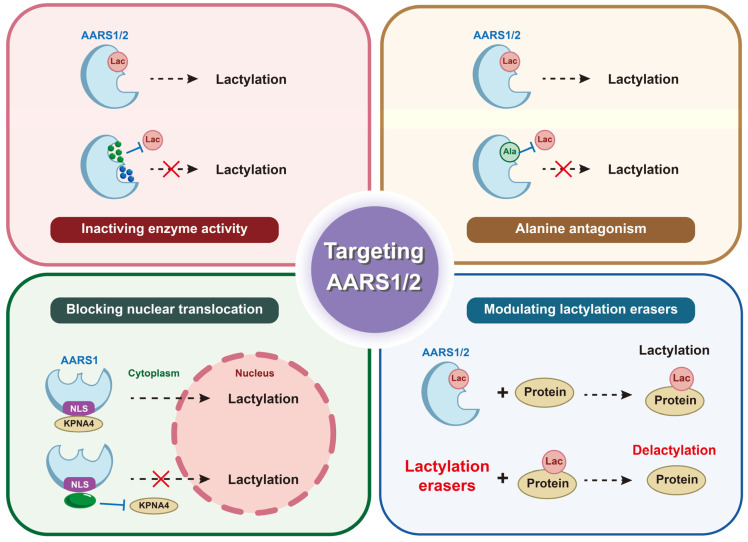
Potential ways of targeting AARS1 and AARS2, including inactivating the enzyme activity of AARS1/2, antagonizing AARS1/2 with alanine, blocking the nuclear translocation of AARS1, and modulating lactylation erasers.

Targeting lactylation erasers is a promising strategy to counteract oncogenic lactylation mediated by AARS1/2. AARS1 was identified as the lactylation “writer”, while histone deacetylase 2 (HDAC2) acted as the “eraser”, removing lactylation from NUDT21, whose overexpression reduced ESCC cells’ resistance to cuproptosis [58]. SIRT1 was reported to delactylate YAP and TEAD1 in gastric cancer [16]. In addition, SIRT1 delactylated canopy FGF signaling regulator 3 (CNPY3) and caused CNPY3 mislocalization, leading to lysosomal rupture, CatB release, caspase-1/GSDMD activation, and pyroptosis in prostate cancer cells [68]. These findings suggest that HDAC2 and SIRT1 act as delactyltransferases and disrupt lactylation-driven oncogenesis. However, challenges exist when activating lactylation erasers to suppress oncogenic lactylation. For instance, HDAC2 was overexpressed in CRC tissues and cell lines, whose high expression correlated with poor prognosis in CRC patients. HDAC2 epigenetically silenced the NLR family pyrin domain containing 3 (NLRP3) gene by deacetylating H3K27ac at its promoter region, preventing pyroptosis in CRC cells treated with antitumor agents like 5-FU or regorafenib [69]. HDAC2, as a deacetylase, promoted pancreatic ductal adenocarcinoma (PDAC) metastasis by sustaining the pro-survival program driven by receptor tyrosine kinases in undifferentiated mesenchymal PDAC cells and protecting mesenchymal PDAC cells from the tumor-suppressive effects of the transforming growth factor β (TGFβ) pathway [70]. HDAC2 promoted tumor progression by silencing pro-apoptotic proteins and inactivating tumor suppressors like p53, whose inhibition by Hit-3 suppressed CRC proliferation and reduced tumor growth [71]. The possible tumor-promoting function of lactylation erasers beyond lactylation should be emphasized, which necessitates achieving cancer cell selectivity and accurate modulation targeting lactylation. Preclinical validation in lactylation-driven oncogenesis by writers like AARS1/2 remains essential to harness the modulation of erasers therapeutically (Figure 4).

## 7. Discussion

### 7.1. Profiling the Full Spectrum of Lactylation Targets of AARS1/2

Profiling the full spectrum of the lactylation targets of both cytoplasmic AARS1 and mitochondrial AARS2 exhibits a profound frontier in understanding how lactate directly regulates protein synthesis as a key metabolic molecular signal. Integrative lactylome and proteome analyses have mapped numerous metabolic enzymes and chromatin regulators as lysine-lactylated substrates in cancers, including gastric cancer, gastrointestinal cancer, hepatocellular carcinoma, and oral squamous cell carcinoma [41,72,73,74,75]. As the roles of AARS1/2 as lactate sensors and lactyltransferases are emerging, comprehensive lactylation profiling specifically for AARS1/AARS2 in cancers is worth exploring, which entails identifying all lactylated lysine sites on AARS1 and AARS2, respectively, and under varying metabolic conditions such as high/low lactate levels or hypoxia/normoxia. Given the different subcellular localizations and metabolic environments of AARS1 and AARS2, their lactylation profiles and functional impacts on cellular biological processes are likely distinct.

### 7.2. Identifying Tissue/Cancer-Type Specificity of AARS1/AARS2 Functions and Lactylation

It is of significance to identify the tissue- or cancer-specific functions of AARS1 and AARS2, extending beyond their canonical roles in protein synthesis, particularly concerning the novel regulatory mechanism of lactylation. AARS1/2 exhibit “moonlighting” functions in various signaling pathways relevant to tumorigenesis. Existing evidence suggests that AARS1/2 catalyze lactylation, but tissue/cancer-specific identification is lacking. The functional impact of AARS1/2-mediated lactylation could be highly context-dependent. Lactate levels, lactyl-CoA availability, and the expression of “writers” and “erasers” of lactylation might vary significantly among tissues, potentially affecting AARS1/2 functions differently. Aggressive, glycolytic tumors that generate immense lactate might provide a favorable microenvironment for AARS1/2-mediated lactylation. Conversely, lactate-poor tumors might have a comparatively small lactylation impact. Therefore, specific identification of AARS1/2 expression and lactylation-related function across different tissues and cancer types is essential to offer novel, context-dependent therapeutic targets for cancer and other diseases driven by AARS1/2 dysregulation.

### 7.3. Crosstalk Between Lactylation and Other PTMs via AARSs

Lactylation entails lactate as a direct lactyl donor, in parallel with the role of acetyl-CoA in acetylation [76]. The underlying mechanism of lactylation is similar to that of acetylation, where lactyl groups antagonize with acetyl groups to occupy the hydroxyl group on lysine. Presumably, lactylation modification overlaps with other PTMs, like acetylation. Normally, the concentration of intracellular lactyl-CoA is hundreds of times lower than that of acetyl-CoAs [43], indicating that acetylation outweighs lactylation in physiological processes. However, in the TME, elevated lactate levels may tip the balance, giving lactylation a significant advantage over acetylation. For instance, acetylation modification can activate p53 tumor-suppressor function [77,78], whereas lactylation modification promotes an oncogenic function [15]. The AARS1-mediated lactylation of p53 antagonizes and outweighs p53 acetylation, thus inhibiting p53 activation [15]. It is a possible avenue to inhibit tumors by regulating the relative concentration of lactate versus acetyl-CoAs and the preference of relevant enzymes toward lactyltransferase activity or acetyltransferase activity. Such a comparative analysis is needed to understand the competitive and cooperative relationships between these modifications. Reversing the oncogenic trend and rebuilding the balance should be taken into consideration when seeking novel antitumor therapies.

Lactylation can impose opposing effects on protein stability via ubiquitination. In gastric cancer, lactylation and ubiquitination can be mutually exclusive and occur in different subcellular compartments. Lactylation occurs primarily in the nucleus and promotes YAP/TEAD1 stability and activity, while ubiquitination occurs in the cytoplasm and targets YAP/TEAD1 for degradation. Lactylation inhibits YAP nuclear export via XPO1 and thus protects YAP/TEAD1 from cytoplasmic ubiquitination [16]. Meanwhile, in bladder cancer, AARS1-mediated lactylation promotes RNF183-mediated ubiquitination and the degradation of YTHDC1. This contrast underscores the context-dependent nature of lactylation and ubiquitination crosstalk, where AARS1 can either facilitate or suppress ubiquitination depending on the substrate, highlighting the complexity of post-translational regulation in tumor biology.

### 7.4. Depicting the Lactylation Profile in Tumor Immunity

The TME comprises not only tumor cells but also immune cells and an extracellular matrix [53,54]. The TME is an immunosuppressive microenvironment, allowing tumor cells to escape immune detection. The accumulation of lactate plays an important role in immune suppression via lactylation modification within immune cells [79], transferring immune cells into an immunosuppressive phenotype [80], generating immunosuppressive cells [81], and promoting the function of immunosuppressive cells [82]. Lactate sensors contribute to global lactylation in immune cells. In macrophages, AARS2 mediates the lactylation of cGAS, leading to impaired enzyme activity of cGAS, reduced GAMP synthesis, and the suppression of innate immune responses [45]. Focusing on the role of lactate sensors in immune cells and tumor immune evasion will provide a novel perspective for tumor therapy.

### 7.5. Highlighting the Role of Lactate Sensors in Tumorigenesis

AARS1/2 are overexpressed in the context of intracellular lactate accumulation, which is closely related to tumorigenesis. There are evolutionarily conserved residues in AARSs that are pivotal to lactate binding. Meanwhile, AARS1/2 act as fundamental lactyltransferases to catalyze global lysine lactylation during tumor progression. Considerably, lactate sensors play an important role in tumor pathophysiology. It is a promising point for cancer therapy to target the lactate sensor from the three perspectives above.

Furthermore, AARS1/2 have been found to bind to lactate and mediate lactylation on the basis of the similarity in chemical structure between lactate and alanine. It is possible that there are other enzymes that act as lactate sensors and lactyltransferases specifically. Further investigations identifying lactylation-related enzymes are required to help understand the mechanism and develop new targets for cancer therapy. Meanwhile, any biological process, including lactylation, is a dynamic balance, indicating that lactylation is mutually in company with delactylation. The underlying mechanism of delactylation and its regulation is unclear and should be brought to the forefront. The deacetylase SIRT1 has been reported to downregulate the lactylation of YAP and TEAD1 [16]. Speculatively, delactylation overlaps with deacetylation, sharing some essential enzymes. Whether lactylation/delactylation overlaps with other PTMs also remains unclear.

### 7.6. Modulating Lactate Sensing via Lactate Production and Transportation

The intracellular lactate concentration depends on the balance between lactate generation and lactate transportation via monocarboxylate transporters (MCTs). Therefore, any factor affecting this balance will ultimately modulate the lactate signal sensed by AARS1/2. The M2 isoform of pyruvate kinase (PKM2) is selectively expressed in cancer cells and is essential for aerobic glycolysis in the Warburg effect, characterized by high lactate production, even in the presence of oxygen. The knockdown of PKM2 and replacement with the adult isoform PKM1 in cancer cell lines reversed aerobic glycolysis, reducing lactate production and increasing oxygen consumption [83]. MCTs, particularly MCT1 and MCT4, are critical for maintaining the high glycolytic flux of cancer cells by preventing intracellular acidification through lactate efflux [84]. The inhibition of MCTs has been shown to cause an accumulation of intracellular lactate [85], thereby amplifying the intracellular lactate signal and potentially activating AARS1/2-dependent pathways. In the context of high PKM2 activity and high MCT expression, where the glycolytic flux is high, lactate is produced and exported efficiently, and it may provide a moderate sensing signal to AARS1/2. This balance, which is coregulated by lactate generation and transportation, fine-tunes the levels of intracellular lactate to suit the tumor’s energetic needs.

### 7.7. Exploring the Regulatory Mechanism of AARS1/2 and Developing Isoform-Specific and Function-Specific Inhibitors

Targeting the regulatory mechanisms controlling the oncogenic functions of AARS1 and AARS2, particularly lactylation-related mechanisms, holds promising therapeutic potential for cancers but requires confronting challenges by developing isoform-specific and function-specific inhibitors. Primary regulatory mechanisms include alternative splicing, which generates functionally distinct variants possessing altered protein expression, enzyme activity, stability, and protein–protein interactions. For example, the knockout of *Pcbp1* in mice leads to the abnormal skipping of exon 16 in *AARS2* pre-mRNA, resulting in a frameshift and premature termination and, consequently, reduced AARS2 protein expression [26]. Additionally, lactylation might potentially modulate AARS1/2 catalytic activity and signaling pathways in return.

Given the high sequence and structure conservation of AARS1/2, developing specific and effective inhibitors is complex, necessitating the identification of subtle structural differences, particularly in the non-catalytic domains acquired during evolution or generated by alternative splicing, to selectively target AARS1 or AARS2 without having mutual effects or disrupting mitochondrial or cytosolic translation. Furthermore, it is promising to develop function-specific regulators that specifically block the functional consequences of lactylation to disrupt lactylation-dependent protein complexes without affecting aminoacylation.

## 8. Conclusions

In summary, this review highlights the novel lactylation-dependent roles of AARS1 and AARS2 in cancers that extend beyond their canonical functions in protein synthesis. Lactylation, as a pivotal post-translational modification, serves as a key mechanistic link between AARS1/2 and the activation of diverse oncogenic signaling pathways. Dysregulation of AARS1/2 contributes to various cancer hallmarks like metabolic reprogramming, uncontrolled proliferation, immune escape, and therapy resistance in a lactylation-driven manner. Targeting AARS1/2 or their lactylation-driven oncogenic signaling pathways represents a promising, novel therapeutic strategy for cancers. Therefore, deepening our molecular understanding of AARS1/2 lactylation profiles and intensifying further research aimed at developing lactylation regulators or specific inhibitors are urgently needed to translate this mechanism into effective cancer therapies.

## Figures and Tables

**Table 1:** The expression and roles of AARS1 and AARS2 in cancers.

Protein	Types of Cancer	Expression in Cancer	Survival and Prognosis	Mechanism and Modification Site	Signaling Pathway	Roles in Cancer	Ref.
AARS1	Bladder cancer	N/A	N/A	K82 lactylation on YTHDC1 protein	YTHDC1-RNF183-JUND-NECTIN4-EV therapy	Reducing cancer cell sensitivity to EV therapy	[57]
AARS1	Breast cancer	Overexpression	Negatively associated	K120 and K139 lactylation on p53 protein	p53 pathway	Promoting cancer cell proliferation	[15]
AARS1	Duodenal cancer	Overexpression	N/A	K621 alanylation on PARP1 protein	DNA damage and cell apoptosis	Suppressing cancer cell apoptosis	[47,63]
AARS1	Esophageal squamous cell carcinoma	N/A	N/A	K23 lactylation on NUDT21 protein	NUDT21-CPSF6-FDX1-cuproptosis	Reducing cancer cells sensitivity to cuproptosis	[58]
AARS1	Gastric cancer	Overexpression	Negatively associated	K90 lactylation on YAP protein, K108 lactylation on TEAD1 protein	Hippo pathway	Promoting cancer cell proliferation	[16]
AARS2	Colon adenocarcinoma	Overexpression	Negatively associated	Regulating mitochondrial respiration	N/A	Promoting cell proliferation	[51]
AARS2	Colorectal cancer	Overexpression	Positively associated	N/A	N/A	N/A	[49]
AARS2	Hepatocellular carcinoma	Overexpression	Negatively associated	Regulating cell cycle	mTOR signaling pathway	Promoting cancer cell proliferation and migration	[50]

## Data Availability

Not applicable.

## Supplementary Materials

The following supporting information can be downloaded at: https://www.mdpi.com/article/10.3390/biom15091323/s1, Figure S1: Alignment of AARS1 protein sequences of different species with Clustal Omega (Version 1.2.2); Figure S2: Alignment of AARS2 protein sequences of different species with Clustal Omega.

## Abbreviations

The following abbreviations are used in this manuscript:

AARS	Aminoacyl-tRNA synthetase
Acetyl-CoA	Acetyl-coenzyme A
Acyl-CoA	Acyl-coenzyme A
AlaRS	Alanyl-tRNA synthetase
ACSL4	Acyl-CoA synthetase long-chain family member 4
AREG	Pro-angiogenic mediator amphiregulin
ARS	Aminoacyl-tRNA synthetase
ATP	Adenosine triphosphate
BC	Bladder cancer
cGAS	Cyclic GMP–AMP synthase
CNPY3	Canopy FGF signaling regulator 3
COAD	Colon adenocarcinoma
CPSF6	Cleavage and polyadenylation specific factor 6
CPT2	Carnitine palmitoyltransferase 2
CRC	Colorectal cancer
CREB	Akt-cAMP response element binding protein
DC	Duodenal cancer
dPAS	Distal polyadenylation site
ESCC	Esophageal squamous cell carcinoma
EV	Enfortumab vedotin
FDX1	Ferredoxin 1
FSH	Follicle-stimulating hormone
HDAC2	Histone deacetylase 2
GC	Gastric cancer
GPR	G-protein-coupled receptor
HCC	Hepatocellular carcinoma
HMGB1	High mobility group box 1
JUND	JunD proto-oncogene
K-AA	Lysine aminoacylation
K-Ala	Lysine alanylation
KPNA4	Karyopherin subunit alpha 4
Lactyl-CoA	Lactyl-coenzyme A
LDHA	Lactate dehydrogenase
LLPS	Liquid–liquid phase separation
MCT	Monocarboxylate transporter protein
NECTIN4	Nectin cell adhesion molecule 4
NLS	Nuclear localization signal
NLRP3	NLR family pyrin domain containing 3
NPS-TTD	Unsolved non-photosensitive trichothiodystrophy
NUDT21	Nudix hydrolase 21
Osx	Osterix
PARP1	Poly (ADP-ribose) polymerase 1
Pcbp1	Poly(rC) binding protein 1
PDAC	Pancreatic ductal adenocarcinoma
PDHA1	Pyruvate dehydrogenase A1
PKM2	Pyruvate kinase M2
PI3K	Phosphoinositide 3-OH kinase
PPARγ	Peroxisome proliferator-activated receptor γ
PPi	Inorganic pyrophosphate
PTM	Post-translational modification
RNF183	Ring finger protein 183
RS	Risk score
SIRT1	Sirtuin 1
TAM	Tumor-associated macrophage
TEAD1	TEA domain transcription factor 1
TFAM	Mitochondrial transcription factor A
TGFβ	Transforming growth factor β
TME	Tumor microenvironment
WDR5	WD repeat domain 5
YAP	Yes1-associated transcriptional regulator
YTHDC1	YTH N6-methyladenosine RNA binding protein C1
