# Supplementary figures and images for: AARS1 and AARS2: From Protein Synthesis to Lactylation-Driven Oncogenesis
Source: Biomolecules. 2025 Sep 16;15(9):1323. doi: 10.3390/biom15091323 (PMC12467997; doi:10.3390/biom15091323)

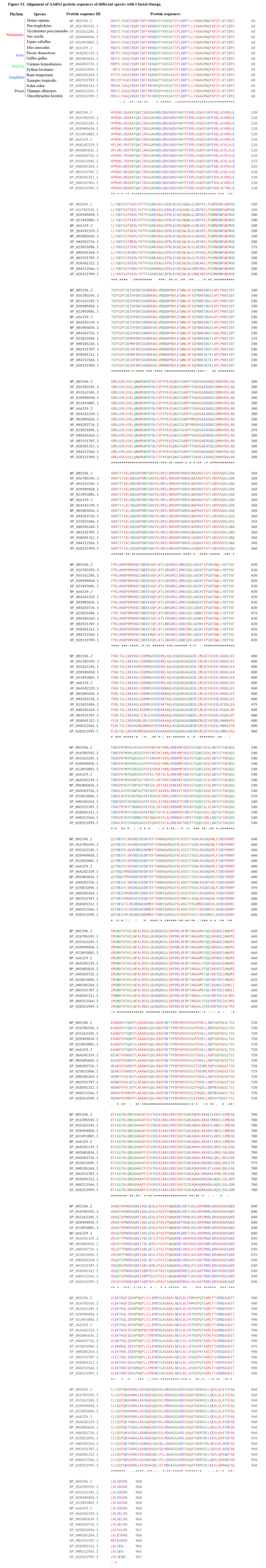

Supplement: Supplementary file 1 [file biomolecules-15-01323-s001.zip › Figure S1.tif]

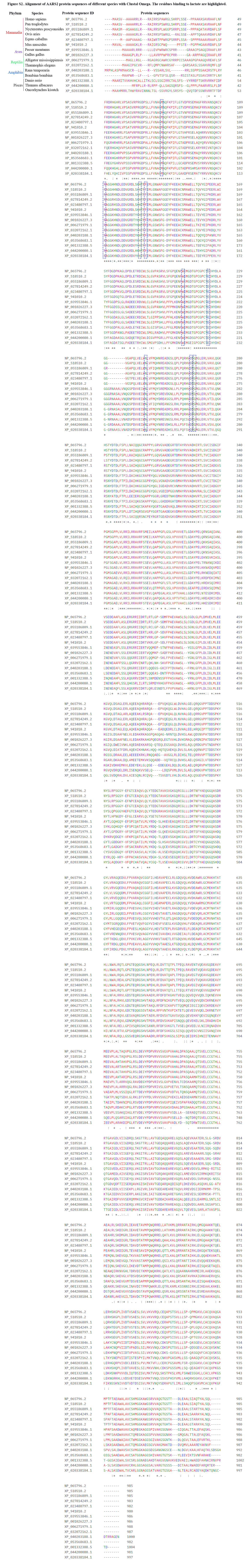

Supplement: Supplementary file 1 [file biomolecules-15-01323-s001.zip › Figure S2.tif]
